# Temporal associations between objectively measured physical activity and depressive symptoms: An experience sampling study

**DOI:** 10.3389/fpsyt.2022.920580

**Published:** 2022-07-18

**Authors:** Yu-Mei Li, Kenn Konstabel, René Mõttus, Sakari Lemola

**Affiliations:** ^1^Department of Psychology, Bielefeld University, Bielefeld, Germany; ^2^National Institute of Health Development, Tallinn, Estonia; ^3^Department of Psychology, University of Tartu, Tartu, Estonia; ^4^Department of Psychology, University of Edinburgh, Edinburgh, United Kingdom; ^5^Department of Psychology, University of Warwick, Coventry, United Kingdom

**Keywords:** experience sampling method (ESM), physical activity, accelerometry, negative affect, positive affect, depressive symptoms, within-individual differences

## Abstract

**Background:**

This study aimed to examine the relationship between the timing of physical activity and within-individual differences in depressive symptoms, positive affect, and negative affect in individuals with different baseline levels of depressive symptoms.

**Methods:**

Experience sampling methodology (ESM) was used to measure real-time depressive symptoms, positive affect, and negative affect in a convenience sample of 78 healthy adults (aged *M* = 25.46 years, *SD* = 6.18; 57 females) five times per day for 14 days. We measured physical activity throughout the 14 days by using activity sensors. Baseline levels of depressive symptoms were assessed with the PHQ-9 to build subgroups with low vs. mild-moderate depressive symptoms.

**Results:**

Physical activity predicted decreased depressive symptom levels, negative affect, and increased positive affect. Associations were stronger for individuals with higher baseline levels of depressive symptoms and for physical activity between 0 and 30 min compared to physical activity between 30 and 180 min before the mood rating. Conversely, levels of depressive symptoms, positive and negative affect did not predict physical activity.

**Limitations:**

The convenience sample may not have been representative of the general population or people with depression. Accelerometers may not have detected some types of physical activities such as bicycling. Causality could not be inferred because of the observational study design.

**Conclusion:**

Individuals with higher levels of depressive symptoms may benefit from physical activity. While the effects were strongest for physical activity immediately before the mood ratings, the effects were in the same direction for up to 3 h before the mood ratings.

## Introduction

The benefits of physical activity for the treatment and prevention of depression have been widely reported. A Cochrane systematic review and meta-analysis of randomized control trials has shown that physical exercise may be a moderately effective treatment of major depressive disorder (1). Exercise interventions may also have acute mood enhancing effects (2) and acutely decrease anxiety symptoms (3) and depressive symptoms (4) in patients with clinical levels of anxiety and depression.

A growing body of research has investigated whether naturally occurring everyday physical activity, such as going for a walk or running to catch a bus, is associated with subsequent mood measured with experience sampling methods (ESM). Most studies measuring physical activity objectively with accelerometers have found improved mood after physical activity (5–12) whereas some have found no association (13, 14).

Similarly, several studies have examined whether mood assessed with ESM is associated with subsequent physical activity as assessed objectively with accelerometers (6, 11, 12, 15–19). Several studies (6, 11, 16, 18) indicated an increase in physical activity when the mood is positive, but three studies (12, 17, 19) could not confirm the association.

However, there are also important gaps in our knowledge on the association between mood and physical activity, which the current ESM study with concurrent continuous activity monitoring with accelerometers aims to address. First, little is known about the role of the time interval between physical activity and mood ratings; that is, how long any effects, if present at all, are sustained. ESM studies for instance specified the time window of measuring physical activity as 15 min before the mood rating (14) or 30 min before and after the mood rating (6). Such a procedure does not allow inspection of whether the effects fade away at some point after 30 min have elapsed. Moreover, an experimental intervention study with patients with major depression even found opposite effects depending on how much time had elapsed after physical activity; examining the course of mood after a treadmill exercise intervention, that study showed positive effects on mood immediately after physical activity while negative effects on mood prevailed 30 min after physical activity (20). To fill this gap in our knowledge, the current study aims to examine associations of physical activity during different time windows with subsequently measured mood as well as associations of mood with subsequent physical activity after varying time intervals. Specifically, we studied the associations with physical activity in 30-min time windows, these are 180–150, 150–120, 120–90, 90–60, 60–30, and 30–0 min before and after mood ratings.

Second, the effects may diverge among participants depending on their vulnerability to experiencing a bad mood. Wichers and colleagues (21) for instance found stronger effects of physical activity on mood in participants without a depression diagnosis compared to participants with a depression diagnosis. By contrast, Mata et al. (22) showed similar strength of the association in participants with and without depressive disorder in a study with self-reported physical activity. As existing evidence is inconsistent on whether the moods of individuals with higher depression levels are more or less linked with physical activity, we divided the participants into two groups based on their baseline levels of depressive symptoms to compare the effect sizes.

Third, there is limited evidence on whether associations of physical activity with subsequent mood are different for positive and negative affect. To our knowledge, only three studies indicated stronger effects of physical activity on positive affect than negative affect (12, 21, 22). From psychology, it is clear that positive and negative effects are not the opposing poles of one dimension (23–26). The current study therefore explores differences in the effect sizes for positive and negative affect. Furthermore, the current study also examines associations between physical activity and depressive symptoms as measured with the ESM, which does not only involve lower levels of positive affect and higher levels of negative affect but also further symptom types such as vegetative (e.g., tiredness) and cognitive symptoms of depression (e.g., difficulty concentrating).

Fourth, there is limited evidence on whether there are differential associations of light physical activity (LPA) vs. moderate-to-vigorous physical activity (MVPA) with mood. A few ESM studies using accelerometers focused on MVPA only and showed that MVPA is associated with improved mood afterward (6, 18), while this association was not confirmed by one study (13). Only one study examined associations of LPA showing that light physical activity was not associated with mood afterward in a group of habitually sedentary students (14). However, to our knowledge, no study has yet examined different intensity levels of objectively assessed physical activity within the same study, which is necessary to determine differential associations with mood and to provide advice on which physical exercise intensity might have stronger effects. In the current study, we therefore explored such differential associations of different intensity levels of physical activity by applying an isotemporal substitution approach. This allows simultaneous analysis of different intensity levels of physical activity and provides an estimate of their unique contribution while controlling the effects of other physical activity intensity levels (27, 28).

## Materials and methods

### Design and procedure

This project was approved by the Psychology Research Ethics Committee at the University of Edinburgh. The study was advertised with E-mails from course organizers and department and school secretaries across the university, posters on the campuses, local cafes and shops, and social media. Participants included University students, staff, and visitors who followed a link to register for the study. After participants signed up for the study, they received an E-mail inviting them to collect an activity sensor at a lab. In the lab, they were debriefed, signed an informed consent form, installed a mobile phone application on their phones, and collected an activity sensor—Movisens EcgMove 3 (29)—and accessories including a chest belt, a charging cable, and 14 alcohol pads. While the EcgMove 3 is also able to record electrocardiography, the current study only reports results on accelerometers. Android phone users installed movisensXS (30) and iOS users installed Qumi (31). The participants who had other types of mobile phones (e.g., Blackberry, Windows, or non-smart phones) or Android or iOS phones with incompatible operating systems were given an Android lab phone to use during their participation. We used 11 sensors and 10 lab phones. The study was conducted October 2017–March 2018.

The 14-day experience-sampling period started a day after the participants installed the mobile application. During the 14 days, the participants completed mobile questionnaires five times a day at random times—the first questionnaires 08:00–11:30 during the weekdays and 09:00–11:30 during the weekends, and the second, third, and fourth questionnaires 13:30–19:00, and the last questionnaires 21:00–23:00 during the weekdays and weekends. The notifications for the daily questionnaires were sent out at random times. There was a minimal gap of 2 h between each questionnaire. During the 14 days, the participants were asked to wear an activity sensor at their chests and underneath their clothes at all times except when they were sleeping, showering, bathing, and doing water sports. To ensure the sensors work properly and record data continuously, the participants were asked to charge the sensors every night and to wash the chest belts in the evenings and dry them overnight. After the 14 days, the participants received another E-mail asking them to complete a final questionnaire, return the activity sensor and the accessories, and collect compensation of £8.

### Participants

In total, 143 participants signed up for the study, 105 participants completed the baseline questionnaire of which 22 did not show up to collect the device or dropped out before starting the ESM questionnaire and 5 had problems with the mobile phone application (Qumi) and could not send their ESM data back to the researcher. The remaining 78 participants were included in the analyses (57 females and 21 males; mean age = 25.46 and *SD* = 6.18), providing data for 4,194 measurement occasions in total.

### Measures

#### Baseline questionnaire

The baseline questionnaire included questions on demographics and the Patient Health Questionnaire (PHQ-9), which was used to measure depression. PHQ-9 is broadly used as a screening tool for depression and has a high internal consistency (Cronbach’s α = 0.91) (32). Various cutoffs from 7 to 15 have been used; we chose to use a cutoff of 7 because it splits our sample into two approximately equal halves, and had reasonably high sensitivity (83%) and specificity (73%) as reported in a meta-analysis (33).

#### ESM questionnaire

The ESM questionnaire was designed to reflect the DSM-V diagnostic criteria of major depressive disorder and contained questions derived from the PHQ-9, the Zung Self-Rating Depression Scale (34), the Hospital Anxiety and Depression Scale (35), and the Depression Anxiety Stress Scales (36). All the questions except the first two questions on bed and wake time are on a 0–100 sliding scale. Six items “*I feel sad*,”, “*I feel irritable*,” “*I am restless*,” “*I feel guilty*,” “*I feel worthless*,” and “*I feel hopeless*” were used to build a score of negative affect (Cronbach’s alpha = 0.86), while two items “*I feel happy*” and “*I enjoy what I am doing*” were used to build a score of positive affect (Cronbach’s alpha = 0.80). The sum of depressive symptoms includes 13 items, i.e., the reversed two positive affect items, the six negative affect items, and the five additional items “*I don’t care about anything*,” “*I am tired*,” “*I am doing things at my normal pace*,” “*I feel that I can’t make decisions*,” and “*I can’t concentrate*” (Cronbach’s α = 0.91).

#### Physical activity

Physical activity was calculated by using three-dimensional movement acceleration, which could distinguish activity types, intensity, frequency, pattern, and duration of activity (37, 38). The acceleration measurement was recorded per minute after the recording started and would not stop until the battery ran out or the sensor was connected to a lab computer. The signals from the three axes were firstly bandpass filtered to remove the offset from gravity and irrelevant high-frequency contents (39). To map the output onto physical activity intensity, we convert the raw acceleration (the unit is in Earth gravity, *g*) to metabolic equivalent of task (MET) by using DataAnalyzer (40). The detailed analyses could be found in movisens online documentation.^1^

For exploratory analyses, physical activity was classified into sedentary behavior, LPA, and MVPA. These are defined based on MET, where sedentary behavior (≤1.5 MET), LPA (>1.5 MET and <3 MET), MPA (≥3 MET and <6 MET), and VPA (≥6 MET).

### Statistical analysis

RStudio (Version 3.6.2) was used in the analyses. Multilevel models were used to test the relationships between physical activity and within-individual differences in positive and negative affect, and the sum of depressive symptoms. The variables were person-mean standardized to remove between-individual variances (41, 42). The package lme4 (Version 1.1-26) was used to fit the multilevel models (43). First, the averages of physical activity recorded within time-windows of 180–150 150–120, 120–90, 90–60, 60–30, or 30–0 min before the participants rated the depressive symptoms was the independent variable and the within-individual differences in positive or negative affect or the sum of depressive symptoms was the outcome variable. In the other half of the models, the within-individual differences in positive or negative affect and the sum of depressive symptoms was the independent variable and the averages of physical activity recorded 0–30, 30–60, 60–90, 90–120, 120–150, or 150–180 min after was the outcome variable. The covariates were age, gender, and prompt number. The identification number of the participants was used as the random intercept. The estimation was restricted maximum likelihood method. Before fitting multilevel models, all continuous variables were grand-mean standardized. To correct for multiple comparison, all *p*-values were corrected by using false discovery rate (44). A conservative significant level of.01 is used. The intraclass correlation coefficients of the grand-mean centered variables are 0.45 for positive affect, 0.69 for both negative affect and depressive symptoms, and 0.03–0.13 for the physical activity variables.

In exploratory analyses, an isotemporal substitution approach was adopted (27, 28) to distinguish the effects of LPA and MVPA. Minutes of sedentary behavior, LPA, and MVPA were aggregated by 30-min time windows before the mood ratings to create total physical activity, and a multilevel model was fit for all participants. Thus, in all models where physical activity predicted mood, minutes of LPA, MVPA, and total physical activity were used instead of raw accelerometer data. In the models, physical activity variables were not standardized (i.e., the original unit—minutes—was preserved), whereas the dependent variables (mood) were standardized. The resulting coefficients can be thus interpreted as the amount of change (in SD units) in mood brought out by substituting 1 unit (minute) of sedentary behavior by the equivalent amount of LPA or MVPA (27).

## Results

Descriptive statistics are displayed in Table 1. In total, 78 participants provided 4,194 experience sampling observations of depressive symptoms, positive, and negative affect. The group with high levels of depressive symptoms at baseline (above cut-off 6/7 of the PHQ-9; *n* = 37; 29 females and 8 males) and the group with low levels of depressive symptoms (below cut-off 6/7 of the PHQ-9; *n* = 41; 28 females and 13 males) provided 1,850 and 2,344 observations, respectively.

**TABLE 1:** Descriptive statistics.

	All participants	High levels of depressive symptoms	Low levels of depressive symptoms
			
	(*N* = 78)	(*n* = 37)	(*n* = 41)
Sex, *n* (%)			
Male	21 (26.92%)	8 (21.62%)	13 (31.71%)
Female	57 (73.08%)	39 (78.38%)	28 (68.29%)
Educational attainment, *n* (%)			
GCSE	2 (2.56%)	0 (0.00%)	2 (4.88%)
A level / Highers	25 (32.05%)	13 (35.14%)	12 (29.27%)
Bachelor’s degree	20 (25.64%)	10 (27.03%)	10 (24.39%)
Master’s degree	24 (30.77%)	10 (27.03%)	14 (34.15%)
PhD	7 (8.97%)	4 (10.81%)	3 (7.32%)
Age, M (SD)	25.46 (6.18)	24.43 (5.45)	26.39 (6.70)
Baseline PHQ-9, *M* (*SD*)	7.91 (5.84)	12.78 (4.87)	3.51 (1.63)
**ESM questionnaire, *M* (*SD*)**			
Positive affect	129.65 (29.61)	118.10 (24.78)	140.07 (30.00)
Negative affect	176.96 (103.18)	228.63 (99.06)	130.33 (83.45)
Depressive symptoms	449.48 (193.76)	547.51 (176.75)	361.02 (165.06)
**Physical activity (milli-g/min), *M* (*SD*)**			
Averaged physical activity 150–180 min before	69.10 (23.65)	63.31 (21.04)	74.33 (24.90)
Averaged physical activity 120–150 min before	71.25 (29.14)	63.72 (27.05)	78.03 (29.60)
Averaged physical activity 90–120 min before	69.56 (26.89)	60.94 (23.62)	77.34 (27.55)
Averaged physical activity 60–90 min before	70.13 (22.79)	66.07 (24.08)	73.80 (21.19)
Averaged physical activity 30–60 min before	68.91 (21.26)	62.96 (21.33)	74.28 (19.96)
Averaged physical activity 0–30 min before	66.03 (21.15)	58.18 (16.73)	73.11 (22.38)
Averaged physical activity 0–30 min after	69.03 (21.81)	61.56 (14.26)	75.78 (25.18)
Averaged physical activity 30–60 min after	68.98 (23.06)	60.89 (17.30)	76.27 (25.28)
Averaged physical activity 60–90 min after	68.34 (25.05)	60.11 (17.24)	75.77 (28.65)
Averaged physical activity 90–120 min after	68.70 (24.44)	61.45 (23.15)	75.24 (23.98)
Averaged physical activity 120–150 min after	69.28 (27.32)	61.89 (24.64)	75.96 (28.17)
Averaged physical activity 150–180 min after	65.48 (24.51)	57.11 (17.79)	73.03 (27.36)
**Physical activity in MET, *M* (*SD*)**			
Average MET 150–180 min before	1.63 (0.23)	1.58 (0.20)	1.67 (0.24)
Average MET 120–150 min before	1.65 (0.27)	1.59 (0.28)	1.70 (0.26)
Average MET 90–120 min before	1.63 (0.24)	1.56 (0.23)	1.69 (0.23)
Average MET 60–90 min before	1.63 (0.20)	1.60 (0.22)	1.66 (0.18)
Average MET 30–60 min before	1.62 (0.19)	1.58 (0.19)	1.67 (0.18)
Average MET 0–30 min before	1.59 (0.21)	1.52 (0.18)	1.66 (0.22)
Average MET 0–30 min after	1.62 (0.21)	1.56 (0.15)	1.68 (0.23)
Average MET 30–60 min after	1.62 (0.21)	1.56 (0.18)	1.68 (0.21)
Average MET 60–90 min after	1.61 (0.22)	1.55 (0.19)	1.67 (0.23)
Average MET 90–120 min after	1.62 (0.22)	1.56 (0.22)	1.68 (0.21)
Average MET 120–150 min after	1.63 (0.25)	1.56 (0.25)	1.69 (0.25)
Average MET 150–180 min after	1.59 (0.23)	1.52 (0.18)	1.66 (0.25)

**FIGURE 1:**
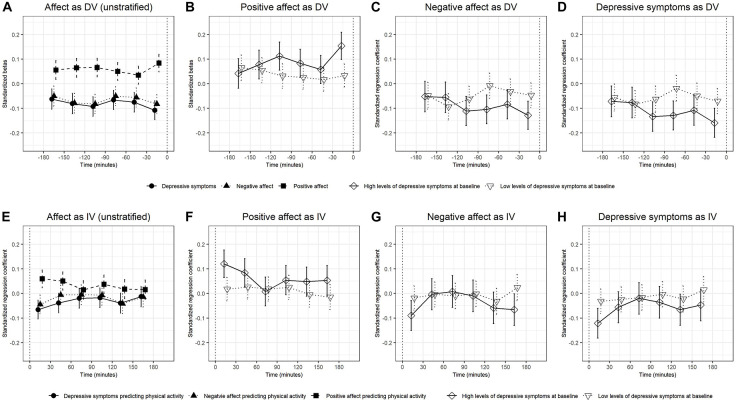
The standardized betas and confidence intervals of multilevel models in which physical activity predicted the subsequently measured dependent variables (DVs) positive affect, negative affect, and depressive symptoms **(A–D)** and models in which the independent variables (IVs) positive affect, negative affect, and depressive symptoms predicted subsequent physical activity **(E–H)**. Results for all participants (unstratified) **(A,E)**. Results for positive affect **(B,F)**, negative affect **(C,G)**, and depressive symptoms **(D,H)** are stratified by baseline levels of depressive symptoms.

Regarding prediction of mood ratings by physical activity, we observed significant associations between physical activity measured in 30-min time windows 3 h before mood ratings and positive and negative affect and the sum of depressive symptoms (Figure 1A and Supplementary Table 1). For positive affect, the effect sizes ranged from 0.03 to 0.08 and the confidence intervals 0.00–0.12. Negative affect and depressive symptoms showed similar effect sizes and confidence intervals; negative affect: −0.05 to −0.08 (standardized beta) and −0.01 to −0.12 (confidence intervals) and depressive symptoms: −0.06 to −0.11 (standardized beta) and −0.02 to −0.15 (confidence intervals).

In the subgroup analyses, the associations of physical activity with positive affect, negative affect, and depressive symptoms, tend to be stronger in the group with higher baseline levels of depressive symptoms (Figures 1B–D and Supplementary Table 2) than in the group with lower baseline levels (Supplementary Table 3). The effects of physical activity on positive affect increased during the 180 min before the mood rating, they were the lowest in the 180–150 min before the mood rating (β = 0.04, CI [−0.02, 0.10]) and the largest in the 30 min before the mood rating (β = 0.15, CI [0.10, 0.21]) and the largest in the 30 min before the mood rating (β = 0.17, CI [0.12, 0.23]). To test whether baseline levels of depressive symptoms moderated the effects, we added a dummy-coded group variable and its interaction with the main predictor to the multilevel models. Significant interactions were observed for physical activity measured 30–0 min before the mood rating predicted positive affect (β = 0.12, CI [0.05, 0.19]) with stronger associations in participants with higher baseline levels of depressive symptoms (Supplementary Table 4; Figure 1).

Regarding prediction of physical activity by mood ratings, significant associations of depressive symptoms, positive affect and negative affect with physical activity measured 0–30 min after the mood ratings were found (Figure 1E). The associations of positive affect (β = 0.12, CI [0.06, 0.18], p < 0.001), negative affect (β = −0.09, CI [−0.15, −0.03], *p* = 0.004), and depressive symptoms (β = −0.12, CI [−0.18, −0.06], *p* < 0.001) with physical activity measured 0–30 min after the mood rating were significant in the group with higher baseline levels of depressive symptoms but they were not significant in the group with lower levels of depressive symptoms (Figures 1F–H).

### Exploratory analysis

**FIGURE 2:**
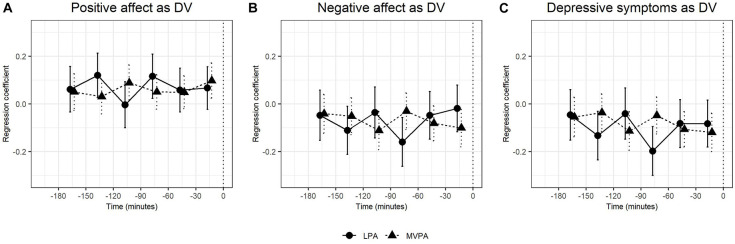
The regression coeffcients and confidence intervals of applying isotemporal substitution models in multilevel models in which light physical activity (LPA), moderate-to-vigorous physical activity (MVPA), and total physical activity (not reported) predicted subsequent positive affect **(A)**, negative affect **(B)**, and depressive symptoms **(C)** in all participants (unstratified). To facilitate better interpretation, the regression coeffcients were multiplied by 10 (thus an unstandardized regression coeffcient of 0.20 means that with every 10-min increase of the respective intensity of physical activity, the dependent variable increases by 20% of a standard deviation).

Applying the isotemporal substitution model (Figure 2; Supplementary Tables 5, 6), LPA measured 90–60 min before the mood ratings significantly predicted negative affect (*b* = −0.02, CI [−0.03, −0.01], *p* = 0.003) and depressive symptoms (*b* = −0.02, CI [−0.03, −0.01], *p* = 0.001).

## Discussion

We found increased physical activity to be associated with increased positive affect, decreased negative affect, and lower levels of depressive symptoms measured up to 180 min after the physical activity was recorded. The size of the association appeared to be relatively constant for physical activity across the 180 min before the mood rating (0.03 to 0.08, −0.05 to −0.08, and −0.06 to −0.11 for positive affect, negative affect, and depressive symptoms, respectively). One exception to this pattern was that in the subgroup analysis of participants with higher baseline depression levels, the effect of physical activity within the last 30 min before the mood ratings on positive affect was larger compared to the effects of physical activity measured 180–30 min before the mood ratings. On average, the associations were more pronounced in participants with increased depressive symptom levels at baseline compared to participants with lower levels of depressive symptoms at baseline and the effect sizes for positive affect, negative affect, and depressive symptom levels were similar. Moreover, exploratory analyses using isotemporal substitution approach showed that there were associations of LPA with subsequent levels of negative affect and depressive symptoms, but we did not detect a meaningful temporal pattern of the effects over the 180 min before the mood rating, which suggests that LPA might have a positive effect on mood.

Regarding the opposite direction of the effect, positive affect, negative affect, and depressive symptom ratings were not associated with subsequent physical activity in most analyses. One notable exception involved acutely decreased physical activity levels in the first 30 min after reporting high levels of negative affect and depressive symptoms as well as increased physical activity after reporting high levels of positive affect, which was particularly pronounced in the group with higher baseline levels of depressive symptoms.

Our findings are generally consistent with previous research showing acute increases in positive affect and/or decreases in negative affect after physical activity (5–12). Our findings go beyond previous research in that they show physical activity was significantly associated with later mood ratings up to 3 h before mood was measured. It has to be noted that physical activity of adjacent 30 min periods is often highly intercorrelated and the coefficients were not adjusted for the effects of the other 30-min periods. It is therefore possible that the physical activity observed more than 30 min before the mood rating was only spuriously related with later mood because of its high correlation with physical activity during the last 30 min before the mood ratings.

Our finding that participants with higher depression levels at baseline appear more susceptible to the effects of physical activity is consistent with Stavrakakis et al. (12) but it is inconsistent with Wichers et al. (21), who found stronger effects in non-depressed participants and Mata et al. (22) who did not find differences in the strength of the association. It has to be noted that in contrast to Stavrakakis et al. (12), Wichers et al. (21), and Mata et al. (22), our study was conducted in young adult university students and depression levels were measured by self-reported questionnaires rather than clinical interviews. Our findings may not apply to people with clinical levels of depression.

Our findings indicate that the effects are relatively similar for positive and negative affect. This is inconsistent with previous studies showing physical activity only had effects on positive affect but not on negative affect (21, 22). Such differences may arise because of differences in the measurement of physical activity in Wichers et al. (21) and Mata et al. (22). It is also possible that this is related to the time interval between physical activity and the mood rating. In our study, the only time window that showed higher effect sizes for positive affect than for negative affect was the last 30 min before the mood ratings, specifically in the subgroup of participants with increased depressive symptom levels at baseline. Though speculative, it is possible that studies that only examine the effects of physical activity immediately before the mood rating in participants with higher depression levels will find stronger effects on positive affect than on negative affect.

Our finding that physical activity was transiently increased in the 30 min after reporting high levels of positive affect and transiently decreased after reporting high levels of negative affect appears to be consistent with an earlier study (16). Kanning and Schoebi (16) showed that greater energetic arousal was associated with increased concurrent physical activity, which, however, decreased over the subsequent 45 min. Importantly, in our study, the effects of mood on subsequent physical activity were on average smaller than effects of physical activity on subsequently measured mood. Therefore, a reverse order of the causal influence appears more plausible for effects of mood on physical activity than for effects of physical activity on mood. However, it has to be acknowledged that causality cannot be shown without an experimental design.

Bossmann et al. (5) have suggested that physical activity prescriptions to improve mood and depressive symptoms may involve electronic reminders encouraging people to engage in frequent daily bouts of physical activity (e.g., going for a walk to interrupt sedentary time). Relatedly, studies using smartphone apps that offer rewards for a larger number of steps made in everyday life suggest that affective wellbeing is also improved (45). In future research, such interventions involving more frequent and shorter bouts of physical activity could be compared with the effectiveness of interventions that involve longer exercising such as 2–3 times a week. Moreover, studies could aim to detect minimal thresholds of physical activity duration, frequency and intensity which are still predictive of subsequent positive mood changes.

Our study suggests that bouts of vigorous physical activity every 3 h might be a promising scheme for such a physical activity intervention in people with increased depressive symptom levels, which could be tested against the effects of different schemes of physical activity.

### Limitations

The findings of our study have to be interpreted in light of the following limitations. First, the participants of this study were relatively young and generally healthy undergraduate students and the findings may not be generalized to other groups defined by different age, social status, physical, and mental health. Second, our findings focus on averaged intra-individual effects while there could be large between-subject differences. Future research may detect different types of people who react differently to physical activity. Third, the accelerometer does not detect all types of physical activity accurately; e.g., vigorous exercising on a treadmill may not have been detected as vigorous physical activity. This may particularly have affected the results of the isotemporal substitution model as the distinction between LPA and MVPA may not have been always accurate. Forth, our study used multilevel models to investigate the relations between physical activity and mood, which also could be studied by using mutual information in future studies.

## Conclusion

Physical activity is associated with improved affect and depressive symptoms up to 3 h after physical activity occurred. This effect was more pronounced in people with higher levels of baseline depressive symptoms, who may be considered more vulnerable to experience negative affective states than those with lower levels of depressive symptoms. Mood specifically predicted subsequent physical activity in the subgroup with higher baseline levels of depressive symptoms and even in this group, the effect vanished after 30 min.

## Data Availability Statement

The raw data supporting the conclusions of this article will be made available by the authors, without undue reservation.

## Ethics statement

The studies involving human participants were reviewed and approved by the Psychology Research Ethics Committee at the University of Edinburgh. Written informed consent from the participants’ legal guardian/next of kin was not required to participate in this study in accordance with the national legislation and the institutional requirements.

## Author contributions

Y-ML: conceptualization, data curation, formal analysis, investigation, software, writing—original draft, visualization, project administration, funding acquisition, writing—review and editing, and methodology. KK: conceptualization, writing—review, visualization, supervision, validation, and methodology. RM: conceptualization, investigation, writing—review, supervision, project administration, funding acquisition, resources, and methodology. SL: conceptualization, writing—review and editing, visualization, supervision, validation, and methodology. All authors contributed to the article and approved the submitted version.

## Conflict of Interest

The authors declare that the research was conducted in the absence of any commercial or financial relationships that could be construed as a potential conflict of interest. The author SL declared that they were an editorial board member of Frontiers, at the time of submission. This had no impact on the peer review process and the final decision.

## Publisher’s Note

All claims expressed in this article are solely those of the authors and do not necessarily represent those of their affiliated organizations, or those of the publisher, the editors and the reviewers. Any product that may be evaluated in this article, or claim that may be made by its manufacturer, is not guaranteed or endorsed by the publisher.

## Footnotes

1
https://docs.movisens.com/Algorithms/energy_expenditure/
