# Corrigendum: Temporal associations between objectively measured physical activity and depressive symptoms: an experience sampling study

**DOI:** 10.3389/fpsyt.2023.1240881

**Published:** 2023-08-23

**Authors:** Yu-Mei Li, Kenn Konstabel, René Mõttus, Sakari Lemola

**Affiliations:** ^1^Department of Psychology, Bielefeld University, Bielefeld, Germany; ^2^National Institute of Health Development, Tallinn, Estonia; ^3^Department of Psychology, University of Tartu, Tartu, Estonia; ^4^Department of Psychology, University of Edinburgh, Edinburgh, United Kingdom; ^5^Department of Psychology, University of Warwick, Coventry, United Kingdom

**Keywords:** experience sampling method (ESM), physical activity, accelerometry, negative affect, positive affect, depressive symptoms, within-individual differences

The following corrections were made because of two issues. First, the daylight-saving time in autumn 2017 and spring 2018 in the sensor data of 15 participants was not adjusted, which resulted in a misalignment of plus or minus one hour between the sensor data and the ESM questionnaire data. The problem arose when data collection ran across daylight-saving time, when a sensor was set up before time change and did not automatically adjust for time change but mobile phones automatically did adjust the time. The sensor data of eight and seven participants were adjusted for the time change in autumn 2017 and spring 2018, respectively. Second, some coding mistakes in the time window calculation were found.

In the published article, there was an error in Table 1 and Figures 1, 2 as published.

In Table 1, the values for physical activity and physical activity in MET were corrected. The values in Figures 1 and 2 were corrected.

The corrected Table 1 and Figures 1, 2 and their captions appear below.

**Table 1:** Descriptive statistics.

	**All participants**	**High levels of depressive symptoms**	**Low levels of depressive symptoms**
	**(*****N*** = **78)**	**(*****n*** = **37)**	**(*****n*** = **41)**
**Sex**, ***n*** **(%)**			
Male	21 (26.92%)	8 (21.62%)	13 (31.71%)
Female	57 (73.08%)	39 (78.38%)	28 (68.29%)
**Educational attainment**, ***n*** **(%)**			
GCSE	2 (2.56%)	0 (0.00%)	2 (4.88%)
A level/highers	25 (32.05%)	13 (35.14%)	12 (29.27%)
Bachelor's degree	20 (25.64%)	10 (27.03%)	10 (24.39%)
Master's degree	24 (30.77%)	10 (27.03%)	14 (34.15%)
PhD	7 (8.97%)	4 (10.81%)	3 (7.32%)
Age, M (SD)	25.46 (6.18)	24.43 (5.45)	26.39 (6.70)
Baseline PHQ-9, *M* (*SD*)	7.91 (5.84)	12.78 (4.87)	3.51 (1.63)
**ESM questionnaire**, ***M*** **(*****SD*****)**			
Positive affect	129.65 (29.61)	118.10 (24.78)	140.07 (30.00)
Negative affect	176.96 (103.18)	228.63 (99.06)	130.33 (83.45)
Depressive symptoms	449.48 (193.76)	547.51 (176.75)	361.02 (165.06)
**Physical activity (milli-g/min)**, ***M*** **(*****SD*****)**			
Average physical activity 150–180 min before	69.10 (23.65)	63.31 (21.04)	74.33 (24.90)
Average physical activity 120–150 min before	71.25 (29.14)	63.72 (27.05)	78.03 (29.60)
Average physical activity 90–120 min before	69.56 (26.89)	60.94 (23.62)	77.34 (27.55)
Average physical activity 60–90 min before	70.13 (22.79)	66.07 (24.08)	73.80 (21.19)
Average physical activity 30–60 min before	68.91 (21.26)	62.96 (21.33)	74.28 (19.96)
Average physical activity 0–30 min before	66.03 (21.15)	58.18 (16.73)	73.11 (22.38)
Average physical activity 0–30 min after	69.03 (21.81)	61.56 (14.26)	75.78 (25.18)
Average physical activity 30–60 min after	68.98 (23.06)	60.89 (17.30)	76.27 (25.28)
Average physical activity 60–90 min after	68.34 (25.05)	60.11 (17.24)	75.77 (28.65)
Average physical activity 90–120 min after	68.70 (24.44)	61.45 (23.15)	75.24 (23.98)
Average physical activity 120–150 min after	69.28 (27.32)	61.89 (24.64)	75.96 (28.17)
Average physical activity 150–180 min after	65.48 (24.51)	57.11 (17.79)	73.03 (27.36)
**Physical activity in MET**, ***M*** **(*****SD*****)**			
Average MET 150–180 min before	1.63 (0.23)	1.58 (0.20)	1.67 (0.24)
Average MET 120–150 min before	1.65 (0.27)	1.59 (0.28)	1.70 (0.26)
Average MET 90–120 min before	1.63 (0.24)	1.56 (0.23)	1.69 (0.23)
Average MET 60–90 min before	1.63 (0.20)	1.60 (0.22)	1.66 (0.18)
Average MET 30–60 min before	1.62 (0.19)	1.58 (0.19)	1.67 (0.18)
Average MET 0–30 min before	1.59 (0.21)	1.52 (0.18)	1.66 (0.22)
Average MET 0–30 min after	1.62 (0.21)	1.56 (0.15)	1.68 (0.23)
Average MET 30–60 min after	1.62 (0.21)	1.56 (0.18)	1.68 (0.21)
Average MET 60–90 min after	1.61 (0.22)	1.55 (0.19)	1.67 (0.23)
Average MET 90–120 min after	1.62 (0.22)	1.56 (0.22)	1.68 (0.21)
Average MET 120–150 min after	1.63 (0.25)	1.56 (0.25)	1.69 (0.25)
Average MET 150–180 min after	1.59 (0.23)	1.52 (0.18)	1.66 (0.25)

**Figure 1:**
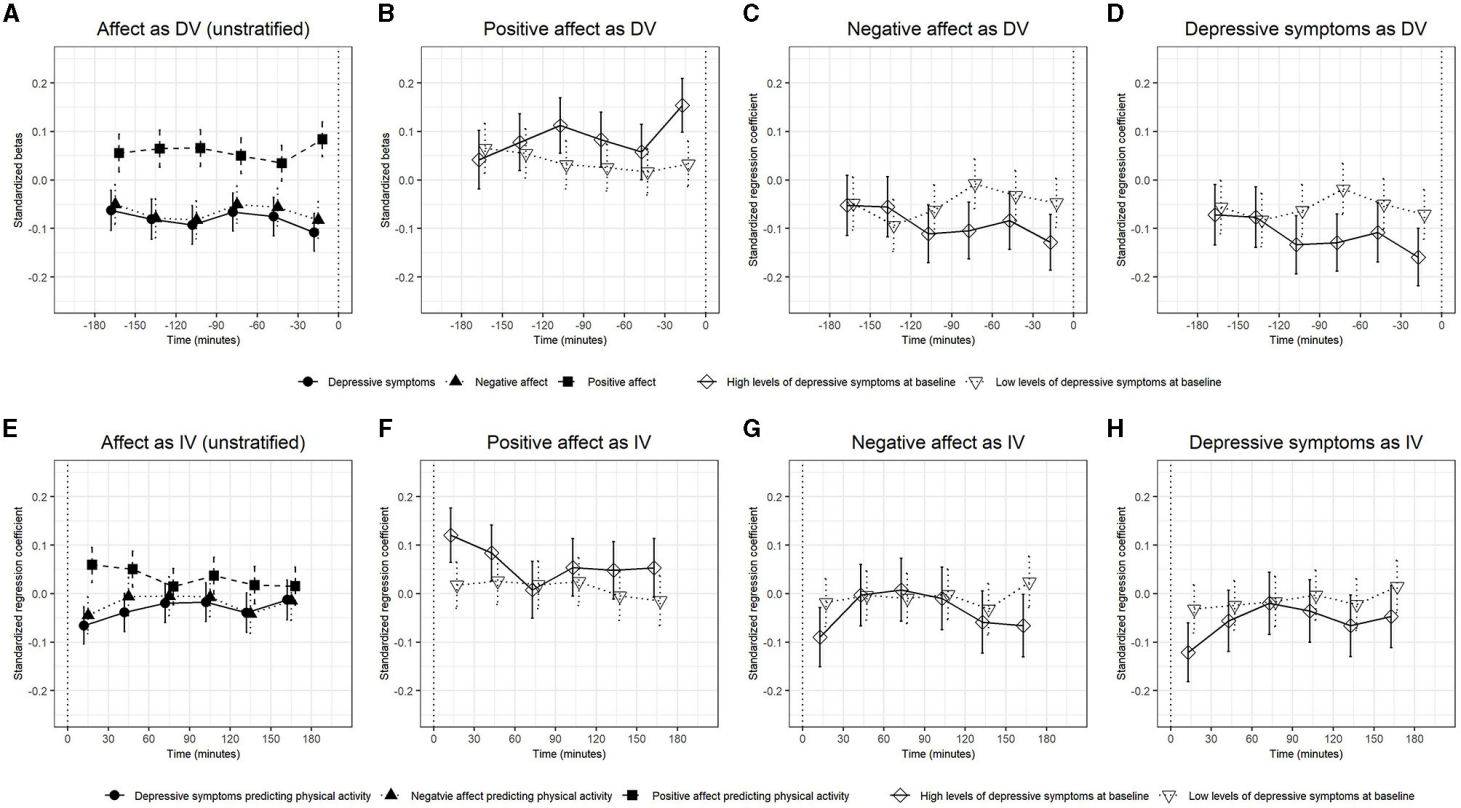
The standardized betas and confidence intervals of multilevel models in which physical activity predicted the subsequently measured dependent variables (DVs) positive affect, negative affect, and depressive symptoms **(A–D)** and models in which the independent variables (IVs) positive affect, negative affect, and depressive symptoms predicted subsequent physical activity **(E–H)**. Results for all participants (unstratified) **(A, E)**. Results for positive affect **(B, F)**, negative affect **(C, G)**, and depressive symptoms **(D, H)** are stratified by baseline levels of depressive symptoms.

**Figure 2:**
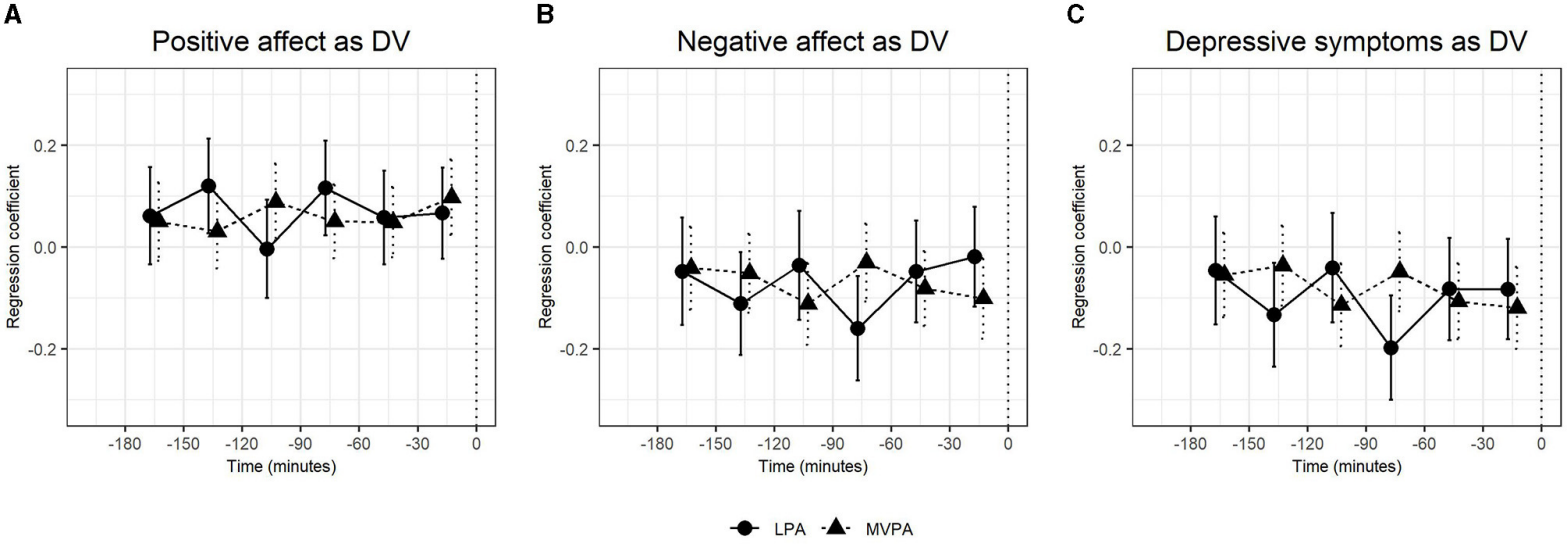
The regression coefficients and confidence intervals of applying isotemporal substitution models in multilevel models in which light physical activity (LPA), moderate-to-vigorous physical activity (MVPA), and total physical activity (not reported) predicted subsequent positive affect **(A)**, negative affect **(B)**, and depressive symptoms **(C)** in all participants (unstratified). To facilitate better interpretation, the regression coefficients were multiplied by 10 (thus an unstandardized regression coefficient of 0.20 means that with every 10-min increase of the respective intensity of physical activity, the dependent variable increases by 20% of a standard deviation).

In the published article, there was an error in Supplementary Tables 1–6 and Supplementary Figure 1. The values in Supplementary Tables 1–6 and Figure S1 were corrected. In the title of Supplementary Table 4, “Only the results of the interaction were presented” was added.

The correct Supplementary material appears below.

**Supplementary Table 1:** Results of multilevel models in which physical activity predicted the subsequent positive affect (PA), negative affect (NA), and depressive symptoms (DS) and the models in which PA, NA, and DS predicted the subsequent physical activity in all participants (N = 78).

	***n***	**β**	***LL***	***UL***	***SE***	***p***
**IV: Physical activity measured 150–180 mins before; DV: PA, NA and DS**						
PA	2565	0.06	0.02	0.09	0.02	.008
NA	2248	−0.05	−0.09	−0.01	0.02	.016
DS	2217	−0.06	−0.10	−0.02	0.02	.008
**IV: Physical activity measured 120–150 mins before; DV: PA, NA and DS**						
PA	2651	0.06	0.03	0.10	0.02	.001
NA	2329	−0.08	−0.12	−0.04	0.02	<.001
DS	2296	−0.08	−0.12	−0.04	0.02	<.001
**IV: Physical activity measured 90–120 mins before; DV: PA, NA and DS**						
PA	2737	0.07	0.03	0.10	0.02	.001
NA	2413	−0.08	−0.12	−0.04	0.02	<.001
DS	2376	−0.09	−0.13	−0.05	0.02	<.001
**IV: Physical activity measured 60–90 mins before; DV: PA, NA and DS**						
PA	2807	0.05	0.01	0.09	0.02	.010
NA	2487	−0.05	−0.09	−0.01	0.02	.010
DS	2446	−0.07	−0.11	−0.03	0.02	.003
**IV: Physical activity measured 30–60 mins before; DV: PA, NA and DS**						
PA	2895	0.03	0.00	0.07	0.02	.066
NA	2570	−0.06	−0.09	−0.02	0.02	.007
DS	2524	−0.08	−0.11	−0.04	0.02	.001
**IV: Physical activity measured 0–30 mins before; DV: PA, NA and DS**						
PA	2987	0.08	0.05	0.12	0.02	<.001
NA	2660	−0.08	−0.12	−0.04	0.02	<.001
DS	2609	−0.11	−0.15	−0.07	0.02	<.001
**IV: PA, NA and DS; DV: Physical activity measured 0–30 mins after**						
PA	2912	0.06	0.02	0.10	0.02	.002
NA	2584	−0.04	−0.08	−0.01	0.02	.021
DS	2536	−0.07	−0.10	−0.03	0.02	.002
**IV: PA, NA and DS; DV: Physical activity measured 30–60 mins after**						
PA	2782	0.05	0.01	0.09	0.02	.023
NA	2456	−0.01	−0.05	0.03	0.02	.760
DS	2407	−0.04	−0.08	0.00	0.02	.079
**IV: PA, NA and DS; DV: Physical activity measured 60–90 mins after**						
PA	2720	0.01	−0.02	0.05	0.02	.672
NA	2397	−0.01	−0.05	0.03	0.02	.794
DS	2347	−0.02	−0.06	0.02	0.02	.672
**IV: PA, NA and DS; DV: Physical activity measured 90–120 mins after**						
PA	2654	0.04	0.00	0.08	0.02	.166
NA	2331	−0.01	−0.05	0.03	0.02	.716
DS	2283	−0.02	−0.06	0.02	0.02	.574
**IV: PA, NA and DS; DV: Physical activity measured 120–150 mins after**						
PA	2602	0.02	−0.02	0.06	0.02	.381
NA	2282	−0.04	−0.08	0.00	0.02	.088
DS	2235	−0.04	−0.08	0.00	0.02	.088
**IV: PA, NA and DS; DV: Physical activity measured 150–180 mins after**						
PA	2499	0.02	−0.02	0.05	0.02	.532
NA	2190	−0.01	−0.06	0.03	0.02	.532
DS	2143	−0.01	−0.05	0.03	0.02	.532

*Note. n* = number of observations; β = standardized beta of the fixed effect; *LL* = lower limit of the confidence interval; *UL* = upper limit of the confidence interval; *SE* = standard error of the fixed effect; *p* = adjusted *p*-value.

**Supplementary Table 2:** Results of multilevel models in which physical activity predicted the subsequent PA, NA, and DS and the models in which PA, NA, and DS predicted the subsequent physical activity in the participants with high levels of depressive symptoms (n = 37).

	***n***	**β**	***LL***	***UL***	***SE***	***P***
**IV: Physical activity measured 150–180 mins before; DV: PA, NA and DS**						
PA	1076	0.04	−0.02	0.10	0.03	.172
NA	941	−0.05	−0.11	0.01	0.03	.149
DS	930	−0.07	−0.13	−0.01	0.03	.072
**IV: Physical activity measured 120–150 mins before; DV: PA, NA and DS**						
PA	1119	0.08	0.02	0.14	0.03	.024
NA	980	−0.06	−0.12	0.01	0.03	.080
DS	967	−0.08	−0.14	−0.01	0.03	.024
**IV: Physical activity measured 90–120 mins before; DV: PA, NA and DS**						
PA	1156	0.11	0.05	0.17	0.03	<.001
NA	1014	−0.11	−0.17	−0.05	0.03	<.001
DS	999	−0.13	−0.19	−0.07	0.03	<.001
**IV: Physical activity measured 60–90 mins before; DV: PA, NA and DS**						
PA	1173	0.08	0.03	0.14	0.03	.004
NA	1032	−0.10	−0.16	−0.05	0.03	.001
DS	1017	−0.13	−0.19	−0.07	0.03	<.001
**IV: Physical activity measured 30–60 mins before; DV: PA, NA and DS**						
PA	1210	0.06	0.00	0.11	0.03	.048
NA	1070	−0.08	−0.14	−0.02	0.03	.009
DS	1053	−0.11	−0.17	−0.05	0.03	.002
**IV: Physical activity measured 0–30 mins before; DV: PA, NA and DS**						
PA	1243	0.15	0.10	0.21	0.03	<.001
NA	1103	−0.13	−0.19	−0.07	0.03	<.001
DS	1085	−0.16	−0.22	−0.10	0.03	<.001
**IV: PA, NA and DS; DV: Physical activity measured 0–30 mins after**						
PA	1203	0.12	0.06	0.18	0.03	<.001
NA	1063	−0.09	−0.15	−0.03	0.03	.004
DS	1050	−0.12	−0.18	0.06	0.03	<.001
**IV: PA, NA and DS; DV: Physical activity measured 30–60 mins after**						
PA	1159	0.08	0.03	0.14	0.03	.013
NA	1020	0.00	−0.07	0.06	0.03	.924
DS	1006	−0.06	−0.12	0.01	0.03	.122
**IV: PA, NA and DS; DV: Physical activity measured 60–90 mins after**						
PA	1135	0.01	−0.05	0.07	0.03	.812
NA	997	0.01	−0.06	0.07	0.03	.812
DS	983	−0.02	−0.08	0.04	0.03	.812
**IV: PA, NA and DS; DV: Physical activity measured 90–120 mins after**						
PA	1108	0.05	−0.01	0.11	0.03	.222
NA	970	−0.01	−0.07	0.05	0.03	.759
DS	956	−0.04	−0.10	0.03	0.03	.413
**IV: PA, NA and DS; DV: Physical activity measured 120–150 mins after**						
PA	1088	0.05	−0.01	0.11	0.03	.113
NA	952	−0.06	−0.12	0.01	0.03	.108
DS	938	−0.07	−0.13	0.00	0.03	.108
**IV: PA, NA and DS; DV: Physical activity measured 150–180 mins after**						
PA	1040	0.05	−0.01	0.11	0.03	.125
NA	908	−0.07	−0.13	0.00	0.03	.125
DS	894	−0.05	−0.11	0.02	0.03	.147

*Note. n* = number of observations; β = standardized beta of the fixed effect; *LL* = lower limit of the confidence interval; *UL* = upper limit of the confidence interval; *SE* = standard error of the fixed effect; *p* = adjusted *p*-value.

**Supplementary Table 3:** Results of multilevel models in which physical activity predicted the subsequent PA, NA, and DS and models in which PA, NA, and DS predicted the subsequent physical activity in the participants with low levels of depressive symptoms (n = 41).

	***n***	**β**	***LL***	***UL***	***SE***	***P***
**IV: Physical activity measured 150–180 mins before; DV: PA, NA and DS**						
PA	1489	0.07	0.01	0.12	0.03	.038
NA	1307	−0.05	−0.10	0.01	0.03	.082
DS	1287	−0.06	−0.11	0.00	0.03	.071
**IV: Physical activity measured 120–150 mins before; DV: PA, NA and DS**						
PA	1532	0.05	0.00	0.11	0.03	.035
NA	1349	−0.09	−0.15	−0.04	0.03	.002
DS	1329	−0.08	−0.14	−0.03	0.03	.005
**IV: Physical activity measured 90–120 mins before; DV: PA, NA and DS**						
PA	1581	0.03	−0.02	0.08	0.03	.213
NA	1399	−0.06	−0.11	−0.01	0.03	.031
DS	1377	−0.06	−0.12	−0.01	0.03	.031
**IV: Physical activity measured 60–90 mins before; DV: PA, NA and DS**						
PA	1634	0.03	−0.02	0.07	0.02	.733
NA	1455	-.01	−0.06	0.04	0.03	.767
DS	1429	−0.02	−0.07	0.03	0.03	.733
**IV: Physical activity measured 30–60 mins before; DV: PA, NA and DS**						
PA	1685	0.02	−0.03	0.06	0.02	.492
NA	1500	−0.03	−0.08	0.02	0.03	.334
DS	1471	−0.05	−0.10	0.00	0.03	.182
**IV: Physical activity measured 0–30 mins before; DV: PA, NA and DS**						
PA	1744	0.03	−0.01	0.08	0.02	.167
NA	1557	−0.05	−0.10	0.00	0.03	.099
DS	1524	−0.07	−0.12	−0.02	0.03	.021
**IV: PA, NA and DS; DV: Physical activity measured 0–30 mins after**						
PA	1709	0.02	−0.03	0.06	0.02	.471
NA	1521	−0.02	−0.07	0.03	0.03	.471
DS	1486	−0.03	−0.08	0.02	0.03	.471
**IV: PA, NA and DS; DV: Physical activity measured 30–60 mins after**						
PA	1623	0.03	−0.02	0.07	0.02	.522
NA	1436	0.00	−0.05	0.05	0.03	.885
DS	1401	−0.02	−0.07	0.03	0.03	.522
**IV: PA, NA and DS; DV: Physical activity measured 60–90 mins after**						
PA	1585	0.02	−0.03	0.07	0.03	.708
NA	1400	−0.01	−0.06	0.04	0.03	.708
DS	1364	−0.02	−0.07	0.04	0.03	.708
**IV: PA, NA and DS; DV: Physical activity measured 90–120 mins after**						
PA	1546	0.02	−0.02	0.07	0.03	.946
NA	1361	0.00	−0.05	0.05	0.03	.946
DS	1327	0.00	−0.05	0.05	0.03	.946
**IV: PA, NA and DS; DV: Physical activity measured 120–150 mins after**						
PA	1514	0.00	−0.06	0.05	0.03	.865
NA	1330	−0.03	−0.08	0.02	0.03	.618
DS	1297	−0.02	−0.08	0.03	0.03	.618
**IV: PA, NA and DS; DV: Physical activity measured 150–180 mins after**						
PA	1459	−0.01	−0.07	0.04	0.03	.594
NA	1282	0.02	−0.03	0.08	0.03	.594
DS	1249	0.01	−0.04	0.07	0.03	.594

*Note. n* = number of observations; β = standardized beta of the fixed effect; *LL* = lower limit of the confidence interval; *UL* = upper limit of the confidence interval; *SE* = standard error of the fixed effect; *p* = adjusted *p*-value.

**Supplementary Table 4:** Results of multilevel models in which physical activity and the interactions between physical activity and low vs. high depressive symptom groups predicted the subsequent PA, NA, and DS, and models in which PA, NA, and DS and the interactions between PA, NA, or DS and low vs. high depressive symptom groups predicted the subsequent physical activity in all participants. Only the results of the interaction were presented.

	***n***	**β**	***LL***	***UL***	***SE***	***P***
**IV: Physical activity measured 150–180 mins before; DV: PA, NA and DS**						
PA	2565	−0.02	−0.10	0.06	0.04	.959
NA	2248	0.00	−0.09	0.08	0.04	.959
DS	2217	−0.02	−0.10	0.07	0.04	.959
**IV: Physical activity measured 120–150 mins before; DV: PA, NA and DS**						
PA	2651	0.02	−0.05	0.10	0.04	.829
NA	2329	0.04	−0.04	0.12	0.04	.829
DS	2296	0.01	−0.08	0.09	0.04	.880
**IV: Physical activity measured 90–120 mins before; DV: PA, NA and DS**						
PA	2737	0.08	0.00	0.16	0.04	.112
NA	2413	−0.05	−0.13	0.03	0.04	.260
DS	2376	−0.07	−0.15	0.01	0.04	.144
**IV: Physical activity measured 60–90 mins before; DV: PA, NA and DS**						
PA	2807	0.06	−0.02	0.13	0.04	.134
NA	2487	−0.10	−0.17	−0.02	0.04	.024
DS	2446	−0.11	−0.19	−0.03	0.04	.020
**IV: Physical activity measured 30–60 mins before; DV: PA, NA and DS**						
PA	2895	0.04	−0.03	0.12	0.04	.279
NA	2570	−0.05	−0.13	0.03	0.04	.279
DS	2524	−0.06	−0.14	0.02	0.04	.279
**IV: Physical activity measured 0–30 mins before; DV: PA, NA and DS**						
PA	2987	0.12	0.05	0.19	0.04	.003
NA	2660	−0.08	−0.16	−0.01	0.04	.036
DS	2609	−0.09	−0.17	−0.01	0.04	.036
**IV: PA, NA and DS; DV: Physical activity measured 0–30 mins after**						
PA	2912	0.10	0.03	0.18	0.04	.018
NA	2584	−0.07	−0.15	0.01	0.04	.074
DS	2536	−0.09	−0.17	−0.01	0.04	.037
**IV: PA, NA and DS; DV: Physical activity measured 30–60 mins after**						
PA	2782	0.06	−0.02	0.13	0.04	.436
NA	2456	0.00	−0.08	0.08	0.04	.993
DS	2407	−0.03	−0.11	0.05	0.04	.680
**IV: PA, NA and DS; DV: Physical activity measured 60–90 mins after**						
PA	2720	−0.01	−0.09	0.06	0.04	.940
NA	2397	0.02	−0.07	0.10	0.04	.940
DS	2347	0.00	−0.09	0.08	0.04	.940
**IV: PA, NA and DS; DV: Physical activity measured 90–120 mins after**						
PA	2654	0.03	−0.05	0.11	0.04	.703
NA	2331	−0.01	−0.09	0.07	0.04	.827
DS	2283	−0.03	−0.11	0.05	0.04	.703
**IV: PA, NA and DS; DV: Physical activity measured 120–150 mins after**						
PA	2602	0.05	−0.03	0.13	0.04	.429
NA	2282	−0.03	−0.11	0.06	0.04	.518
DS	2235	−0.05	−0.13	0.04	0.04	.429
**IV: PA, NA and DS; DV: Physical activity measured 150–180 mins after**						
PA	2499	0.07	−0.01	0.15	0.04	.148
NA	2190	−0.09	−0.17	−0.01	0.04	.103
DS	2143	−0.06	−0.15	0.02	0.04	.148

*Note. n* = number of observations; β = standardized beta of the fixed effect; *LL* = lower limit of the confidence interval; *UL* = upper limit of the confidence interval; *SE* = standard error of the fixed effect; *p* = adjusted *p*-value.

**Supplementary Table 5:** Results of multilevel models in which light physical activity (LPA), moderate-to-vigorous physical activity (MVPA), and total physical activity (TPA) predicted the subsequent PA, NA, and DS in all participants (N = 78). Only the results of LPA were presented.

	***n***	**β**	***LL***	***UL***	***SE***	***P***
**IV: Physical activity measured 150–180 mins before; DV: PA, NA and DS**						
PA	4079	0.01	0.00	0.02	0.00	.390
NA	3690	0.00	−0.02	0.01	0.01	.390
DS	3616	0.00	−0.02	0.01	0.01	.390
**IV: Physical activity measured 120–150 mins before; DV: PA, NA and DS**						
PA	4078	0.01	0.00	0.02	0.00	.018
NA	3689	−0.01	−0.02	0.00	0.01	.031
DS	3615	−0.01	−0.02	0.00	0.01	.018
**IV: Physical activity measured 90–120 mins before; DV: PA, NA and DS**						
PA	4077	0.00	−0.01	0.01	0.00	.938
NA	3688	0.00	−0.01	0.01	0.01	.759
DS	3614	0.00	−0.01	0.01	0.01	.759
**IV: Physical activity measured 60–90 mins before; DV: PA, NA and DS**						
PA	4074	0.01	0.00	0.02	0.00	.015
NA	3686	−0.02	−0.03	−0.01	0.01	.003
DS	3612	−0.02	−0.03	−0.01	0.01	.001
**IV: Physical activity measured 30–60 mins before; DV: PA, NA and DS**						
PA	4074	0.01	0.00	0.02	0.00	.325
NA	3685	0.00	−0.01	0.01	0.01	.349
DS	3611	−0.01	−0.02	0.00	0.01	.321
**IV: Physical activity measured 0–30 mins before; DV: PA, NA and DS**						
PA	4071	0.01	0.00	0.02	0.00	.220
NA	3682	0.00	−0.01	0.01	0.00	.698
DS	3608	−0.01	−0.02	0.00	0.01	.220

*Note. n* = number of observations; β = standardized beta of the fixed effect; *LL* = lower limit of the confidence interval; *UL* = upper limit of the confidence interval; *SE* = standard error of the fixed effect; *p* = adjusted *p*-value.

**Supplementary Table 6:** Results of multilevel models in which light physical activity (LPA), moderate-to-vigorous physical activity (MVPA), and total physical activity (TPA) predicted the subsequent PA, NA, and DS in all participants (N = 78). Only the results of MVPA were presented.

	***n***	**β**	***LL***	***UL***	***SE***	***P***
**IV: Physical activity measured 150–180 mins before; DV: PA, NA and DS**						
PA	4079	0.00	0.00	0.01	0.00	.308
NA	3690	0.00	−0.01	0.00	0.00	.318
DS	3616	−0.01	−0.01	0.00	0.00	.308
**IV: Physical activity measured 120–150 mins before; DV: PA, NA and DS**						
PA	4078	0.00	0.00	0.01	0.00	.415
NA	3689	−0.01	−0.01	0.00	0.00	.415
DS	3615	0.00	−0.01	0.00	0.00	.415
**IV: Physical activity measured 90–120 mins before; DV: PA, NA and DS**						
PA	4077	0.01	0.00	0.02	0.00	.022
NA	3688	−0.01	−0.02	0.00	0.00	.010
DS	3614	−0.01	−0.02	0.00	0.00	.010
**IV: Physical activity measured 60–90 mins before; DV: PA, NA and DS**						
PA	4074	0.00	0.00	0.01	0.00	.330
NA	3686	0.00	−0.01	0.00	0.00	.433
DS	3612	0.00	−0.01	0.00	0.00	.330
**IV: Physical activity measured 30–60 mins before; DV: PA, NA and DS**						
PA	4074	0.00	0.00	0.01	0.00	.169
NA	3685	−0.01	−0.02	0.00	0.00	.046
DS	3611	−0.01	−0.02	0.00	0.00	.015
**IV: Physical activity measured 0–30 mins before; DV: PA, NA and DS**						
PA	4071	0.01	0.00	0.02	0.00	.013
NA	3682	−0.01	−0.02	0.00	0.00	.013
DS	3608	−0.01	−0.02	0.00	0.00	.011

*Note. n* = number of observations; β = standardized beta of the fixed effect; *LL* = lower limit of the confidence interval; *UL* = upper limit of the confidence interval; *SE* = standard error of the fixed effect; *p* = adjusted *p*-value.

**Suppelementary Figure 1:**
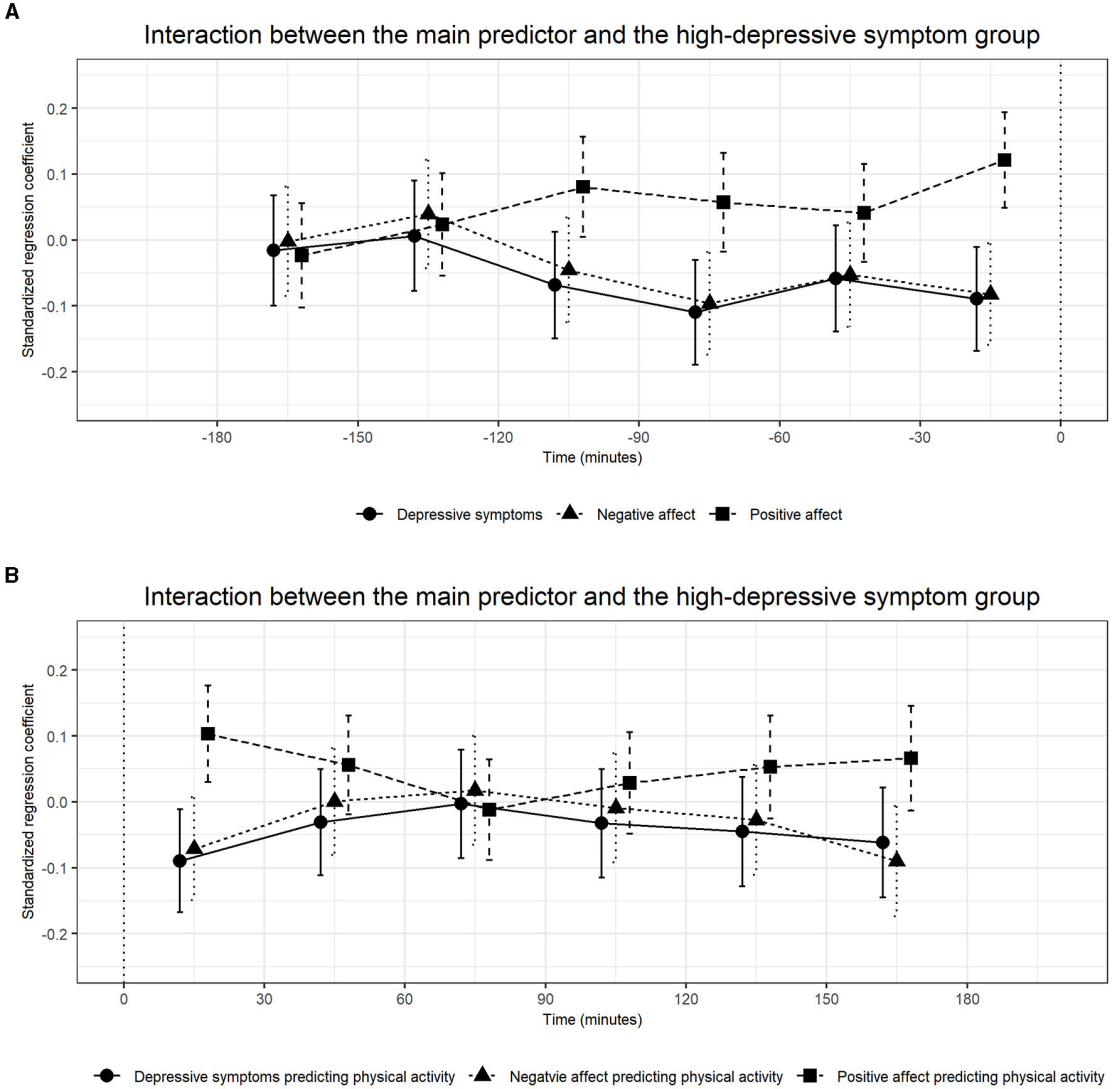
The standardized betas and confidence intervals of multilevel models including an interaction between the main predictor and a dummy-coded group variable.

In the published article, there were errors in **Materials and methods**.

A correction has been made to **Materials and methods**, *Statistical analysis*, paragraph 1. This sentence previously stated:

“The intraclass correlation coefficients of the grand-mean centered variables are 0.45 for positive affect, 0.69 for both negative affect and depressive symptoms, and 0.03–0.15 for the physical activity variables.”

The corrected sentence appears below:

“The intraclass correlation coefficients of the grand-mean centered variables are 0.45 for positive affect, 0.69 for both negative affect and depressive symptoms, and 0.03–0.13 for the physical activity variables.”

A correction has been made to **Materials and methods**, *Statistical analysis*, paragraph 2. This sentence previously stated:

“Minutes of sedentary behavior, LPA, MVPA, and total physical activity were aggregated by 30-min time windows before the mood ratings…”

The corrected sentence appears below:

“Minutes of sedentary behavior, LPA, and MVPA were aggregated by 30-min time windows before the mood ratings to create total physical activity…”

A correction has been made to **Results**, paragraph 2. This sentence previously stated:

“For positive affect, the effect sizes ranged from 0.05 to 0.10 and the confidence intervals 0.01–0.13. Negative affect and depressive symptoms showed similar effect sizes and confidence intervals; negative affect:−0.06 to−0.09 (standardized beta) and−0.02 to−0.13 (confidence intervals) and depressive symptoms:−0.07 to−0.11 (standardized beta) and−0.03 to−0.15 (confidence intervals).”

The corrected sentence appears below:

“For positive affect, the effect sizes ranged from 0.03 to 0.08 and the confidence intervals 0.00–0.12. Negative affect and depressive symptoms showed similar effect sizes and confidence intervals; negative affect:−0.05 to−0.08 (standardized beta) and −0.01 to −0.12 (confidence intervals) and depressive symptoms: −0.06 to −0.11 (standardized beta) and −0.02 to −0.15 (confidence intervals).”

A correction has been made to **Results**, paragraph 3. These sentences previously stated:

“The effects of physical activity on positive affect increased during the 180 min before the mood rating, they were the lowest in the 180–150 min before the mood rating (β = 0.06, CI [0.00, 0.12]) and the largest in the 30 min before the mood rating (β = 0.17, CI [0.12, 0.23]).”

“Significant interactions were observed for physical activity measured 30–0 min before the mood rating predicted positive affect (β = 0.13, CI [0.06, 0.21]) with stronger associations in participants with higher baseline levels of depressive symptoms (Supplementary Table 4; Figure 1).”

The corrected sentence appears below:

“The effects of physical activity on positive affect increased during the 180 min before the mood rating, they were the lowest in the 180–150 min before the mood rating (β = 0.04, CI [−0.02, 0.10]) and the largest in the 30 min before the mood rating (β = 0.15, CI [0.10, 0.21]).”

“Significant interactions were observed for physical activity measured 30–0 min before the mood rating predicted positive affect (β = 0.12, CI [0.05, 0.19]) with stronger associations in participants with higher baseline levels of depressive symptoms (Supplementary Table 4; Figure 1).”

A correction has been made to **Results**, paragraph 4. This sentence previously stated:

“The associations of positive affect (β = 0.13, CI [0.08, 0.19], *p* < 0.001), negative affect (β = −0.10, CI [-0.16,−0.04], *p* = 0.001), and depressive symptoms (β = −0.13, CI [-0.19,−0.07], *p* < 0.001) with physical activity measured 0–30 min after the mood rating were significant in the group with higher baseline levels of depressive symptoms but they were not significant in the group with lower levels of depressive symptoms (Figures 1F–H).”

The corrected sentence appears below:

“The associations of positive affect (β = 0.12, CI [0.06, 0.18], *p* < 0.001), negative affect (β = −0.09, CI [−0.15, −0.03], *p* = 0.004), and depressive symptoms (β = −0.12, CI [−0.18, −0.06], *p* < 0.001) with physical activity measured 0–30 min after the mood rating were significant in the group with higher baseline levels of depressive symptoms but they were not significant in the group with lower levels of depressive symptoms (Figures 1F–H).”

A correction has been made to **Results**, *Exploratory analysis*, paragraph 1. These sentences previously stated:

“Applying the isotemporal substitution model (Figure 2 and Supplementary Tables 5, 6), LPA measured 90–60 min before the mood ratings significantly predicted positive affect (*b* = 0.01, CI [0.00, 0.02], *p* = 0.009), negative affect (*b* = −0.01, CI [-0.02, 0.00], *p* = 0.005) and depressive symptoms (*b* = −0.02, CI [-0.03,−0.01], *p* = 0.003). MVPA measured 30–0 min before the mood rating significantly predicted positive affect (*b* = 0.01, CI [0.00, 0.02], *p* = 0.006) and depressive symptoms (*b* = −0.01, CI [-0.02, 0.00], *p* = 0.006).”

The corrected sentence appears below:

“Applying the isotemporal substitution model (Figure 2; Supplementary Tables 5, 6), LPA measured 90–60 min before the mood ratings significantly predicted negative affect (*b* = −0.02, CI [−0.03, −0.01], *p* = 0.003) and depressive symptoms (*b* = −0.02, CI [−0.03, −0.01], *p* = 0.001).”

A correction has been made to **Discussion**, paragraph 1. These sentences previously stated:

“The size of the association appeared to be relatively constant for physical activity across the 180 min before the mood rating (0.05–0.10,−0.06 to−0.09, and−0.07 to−0.11 for positive affect, negative affect, and depressive symptoms, respectively).”

“Moreover, exploratory analyses using isotemporal substitution approach showed that there were associations of both MVPA and LPA with subsequent levels of positive affect, negative affect and depressive symptoms, but we did not detect stronger effects of MVPA compared to LPA on average nor a meaningful temporal pattern of the effects over the 180 min before the mood rating, which suggests that both MVPA and LPA might both have positive effects on mood.”

The corrected sentences appear below:

“The size of the association appeared to be relatively constant for physical activity across the 180 min before the mood rating (0.03 to 0.08, −0.05 to −0.08, and −0.06 to −0.11 for positive affect, negative affect, and depressive symptoms, respectively).”

“Moreover, exploratory analyses using isotemporal substitution approach showed that there were associations of LPA with subsequent levels of negative affect and depressive symptoms, but we did not detect a meaningful temporal pattern of the effects over the 180 min before the mood rating, which suggests that LPA might have a positive effect on mood.”

The authors apologize for these errors and state that these do not change the scientific conclusions of the article in a substantial manner. However, the significant associations between light physical activity (LPA) measured 90–60 min before the mood ratings and positive affect and between moderate and vigorous physical activity (MVPA) measured 30–0 min before the mood ratings and positive affect and depressive symptoms were no longer significant. The original article has been updated.

