# Supplementary material for: Temporal associations between objectively measured physical activity and depressive symptoms: An experience sampling study
Source: Front Psychiatry. 2022 Jul 18;13:920580. doi: 10.3389/fpsyt.2022.920580 (PMC9339893; doi:10.3389/fpsyt.2022.920580)
Supplement: Supplementary file 1 [file Data_Sheet_1.docx]

**Supplementary Table 1**

*Results of multilevel models in which physical activity predicted the subsequent positive affect (PA), negative affect (NA), and depressive symptoms (DS) and the models in which PA, NA, and DS predicted the subsequent physical activity in all participants (N = 78)*

|  | *n* | β | *LL* | *UL* | *SE* | *p* |
| --- | --- | --- | --- | --- | --- | --- |
| IV: Physical activity measured 150–180 mins before; DV: PA, NA and DS | | | | | | |
| PA | 2565 | 0.06 | 0.02 | 0.09 | 0.02 | .008 |
| NA | 2248 | -0.05 | -0.09 | -0.01 | 0.02 | .016 |
| DS | 2217 | -0.06 | -0.10 | -0.02 | 0.02 | .008 |
| IV: Physical activity measured 120–150 mins before; DV: PA, NA and DS | | | | | | |
| PA | 2651 | 0.06 | 0.03 | 0.10 | 0.02 | .001 |
| NA | 2329 | -0.08 | -0.12 | -0.04 | 0.02 | < .001 |
| DS | 2296 | -0.08 | -0.12 | -0.04 | 0.02 | < .001 |
| IV: Physical activity measured 90–120 mins before; DV: PA, NA and DS | | | | | | |
| PA | 2737 | 0.07 | 0.03 | 0.10 | 0.02 | .001 |
| NA | 2413 | -0.08 | -0.12 | -0.04 | 0.02 | < .001 |
| DS | 2376 | -0.09 | -0.13 | -0.05 | 0.02 | < .001 |
| IV: Physical activity measured 60–90 mins before; DV: PA, NA and DS | | | | | | |
| PA | 2807 | 0.05 | 0.01 | 0.09 | 0.02 | .010 |
| NA | 2487 | -0.05 | -0.09 | -0.01 | 0.02 | .010 |
| DS | 2446 | -0.07 | -0.11 | -0.03 | 0.02 | .003 |
| IV: Physical activity measured 30–60 mins before; DV: PA, NA and DS | | | | | | |
| PA | 2895 | 0.03 | 0.00 | 0.07 | 0.02 | .066 |
| NA | 2570 | -0.06 | -0.09 | -0.02 | 0.02 | .007 |
| DS | 2524 | -0.08 | -0.11 | -0.04 | 0.02 | .001 |
| IV: Physical activity measured 0–30 mins before; DV: PA, NA and DS | | | | | | |
| PA | 2987 | 0.08 | 0.05 | 0.12 | 0.02 | < .001 |
| NA | 2660 | -0.08 | -0.12 | -0.04 | 0.02 | < .001 |
| DS | 2609 | -0.11 | -0.15 | -0.07 | 0.02 | < .001 |
| IV: PA, NA and DS; DV: Physical activity measured 0–30 mins after | | | | | | |
| PA | 2912 | 0.06 | 0.02 | 0.10 | 0.02 | .002 |
| NA | 2584 | -0.04 | -0.08 | -0.01 | 0.02 | .021 |
| DS | 2536 | -0.07 | -0.10 | -0.03 | 0.02 | .002 |
| IV: PA, NA and DS; DV: Physical activity measured 30–60 mins after | | | | | | |
| PA | 2782 | 0.05 | 0.01 | 0.09 | 0.02 | .023 |
| NA | 2456 | -0.01 | -0.05 | 0.03 | 0.02 | .760 |
| DS | 2407 | -0.04 | -0.08 | 0.00 | 0.02 | .079 |
| IV: PA, NA and DS; DV: Physical activity measured 60–90 mins after | | | | | | |
| PA | 2720 | 0.01 | -0.02 | 0.05 | 0.02 | .672 |
| NA | 2397 | -0.01 | -0.05 | 0.03 | 0.02 | .794 |
| DS | 2347 | -0.02 | -0.06 | 0.02 | 0.02 | .672 |
| IV: PA, NA and DS; DV: Physical activity measured 90–120 mins after | | | | | | |
| PA | 2654 | 0.04 | 0.00 | 0.08 | 0.02 | .166 |
| NA | 2331 | -0.01 | -0.05 | 0.03 | 0.02 | .716 |
| DS | 2283 | -0.02 | -0.06 | 0.02 | 0.02 | .574 |
| IV: PA, NA and DS; DV: Physical activity measured 120–150 mins after | | | | | | |
| PA | 2602 | 0.02 | -0.02 | 0.06 | 0.02 | .381 |
| NA | 2282 | -0.04 | -0.08 | 0.00 | 0.02 | .088 |
| DS | 2235 | -0.04 | -0.08 | 0.00 | 0.02 | .088 |
| IV: PA, NA and DS; DV: Physical activity measured 150–180 mins after | | | | | | |
| PA | 2499 | 0.02 | -0.02 | 0.05 | 0.02 | .532 |
| NA | 2190 | -0.01 | -0.06 | 0.03 | 0.02 | .532 |
| DS | 2143 | -0.01 | -0.05 | 0.03 | 0.02 | .532 |

*Note. n* = number of observations; *β* = standardized beta of the fixed effect; *LL* = lower limit of the confidence interval; *UL* = upper limit of the confidence interval; *SE* = standard error of the fixed effect; *p* = adjusted *p*-value.

**Supplementary Table 2**

*Results of multilevel models in which physical activity predicted the subsequent PA, NA, and DS and the models in which PA, NA, and DS predicted the subsequent physical activity in the participants with high levels of depressive symptoms (n = 37).*

|  | *n* | β | *LL* | *UL* | *SE* | *P* |
| --- | --- | --- | --- | --- | --- | --- |
| IV: Physical activity measured 150–180 mins before; DV: PA, NA and DS | | | | | | |
| PA | 1076 | 0.04 | -0.02 | 0.10 | 0.03 | .172 |
| NA | 941 | -0.05 | -0.11 | 0.01 | 0.03 | .149 |
| DS | 930 | -0.07 | -0.13 | -0.01 | 0.03 | .072 |
| IV: Physical activity measured 120–150 mins before; DV: PA, NA and DS | | | | | | |
| PA | 1119 | 0.08 | 0.02 | 0.14 | 0.03 | .024 |
| NA | 980 | -0.06 | -0.12 | 0.01 | 0.03 | .080 |
| DS | 967 | -0.08 | -0.14 | -0.01 | 0.03 | .024 |
| IV: Physical activity measured 90–120 mins before; DV: PA, NA and DS | | | | | | |
| PA | 1156 | 0.11 | 0.05 | 0.17 | 0.03 | < .001 |
| NA | 1014 | -0.11 | -0.17 | -0.05 | 0.03 | <.001 |
| DS | 999 | -0.13 | -0.19 | -0.07 | 0.03 | < .001 |
| IV: Physical activity measured 60–90 mins before; DV: PA, NA and DS | | | | | | |
| PA | 1173 | 0.08 | 0.03 | 0.14 | 0.03 | .004 |
| NA | 1032 | -0.10 | -0.16 | -0.05 | 0.03 | .001 |
| DS | 1017 | -0.13 | -0.19 | -0.07 | 0.03 | < .001 |
| IV: Physical activity measured 30–60 mins before; DV: PA, NA and DS | | | | | | |
| PA | 1210 | 0.06 | 0.00 | 0.11 | 0.03 | .048 |
| NA | 1070 | -0.08 | -0.14 | -0.02 | 0.03 | .009 |
| DS | 1053 | -0.11 | -0.17 | -0.05 | 0.03 | .002 |
| IV: Physical activity measured 0–30 mins before; DV: PA, NA and DS | | | | | | |
| PA | 1243 | 0.15 | 0.10 | 0.21 | 0.03 | < .001 |
| NA | 1103 | -0.13 | -0.19 | -0.07 | 0.03 | < .001 |
| DS | 1085 | -0.16 | -0.22 | -0.10 | 0.03 | < .001 |
| IV: PA, NA and DS; DV: Physical activity measured 0–30 mins after | | | | | | |
| PA | 1203 | 0.12 | 0.06 | 0.18 | 0.03 | < .001 |
| NA | 1063 | -0.09 | -0.15 | -0.03 | 0.03 | .004 |
| DS | 1050 | -0.12 | -0.18 | -0.06 | 0.03 | < .001 |
| IV: PA, NA and DS; DV: Physical activity measured 30–60 mins after | | | | | | |
| PA | 1159 | 0.08 | 0.03 | 0.14 | 0.03 | .013 |
| NA | 1020 | 0.00 | -0.07 | 0.06 | 0.03 | .924 |
| DS | 1006 | -0.06 | -0.12 | 0.01 | 0.03 | .122 |
| IV: PA, NA and DS; DV: Physical activity measured 60–90 mins after | | | | | | |
| PA | 1135 | 0.01 | -0.05 | 0.07 | 0.03 | .812 |
| NA | 997 | 0.01 | -0.06 | 0.07 | 0.03 | .812 |
| DS | 983 | -0.02 | -0.08 | 0.04 | 0.03 | .812 |
| IV: PA, NA and DS; DV: Physical activity measured 90–120 mins after | | | | | | |
| PA | 1108 | 0.05 | -0.01 | 0.11 | 0.03 | .222 |
| NA | 970 | -0.01 | -0.07 | 0.05 | 0.03 | .759 |
| DS | 956 | -0.04 | -0.10 | 0.03 | 0.03 | .413 |
| IV: PA, NA and DS; DV: Physical activity measured 120–150 mins after | | | | | | |
| PA | 1088 | 0.05 | -0.01 | 0.11 | 0.03 | .113 |
| NA | 952 | -0.06 | -0.12 | 0.01 | 0.03 | .108 |
| DS | 938 | -0.07 | -0.13 | 0.00 | 0.03 | .108 |
| IV: PA, NA and DS; DV: Physical activity measured 150–180 mins after | | | | | | |
| PA | 1040 | 0.05 | -0.01 | 0.11 | 0.03 | .125 |
| NA | 908 | -0.07 | -0.13 | 0.00 | 0.03 | .125 |
| DS | 894 | -0.05 | -0.11 | 0.02 | 0.03 | .147 |

*Note. n* = number of observations; *β* = standardized beta of the fixed effect; *LL* = lower limit of the confidence interval; *UL* = upper limit of the confidence interval; *SE* = standard error of the fixed effect; *p* = adjusted *p*-value.

**Supplementary Table 3**

*Results of multilevel models in which physical activity predicted the subsequent PA, NA, and DS and models in which PA, NA, and DS predicted the subsequent physical activity in the participants with low levels of depressive symptoms (n = 41).*

|  | *n* | β | *LL* | *UL* | *SE* | *P* |
| --- | --- | --- | --- | --- | --- | --- |
| IV: Physical activity measured 150–180 mins before; DV: PA, NA and DS | | | | | | |
| PA | 1489 | 0.07 | 0.01 | 0.12 | 0.03 | .038 |
| NA | 1307 | -0.05 | -0.10 | 0.01 | 0.03 | .082 |
| DS | 1287 | -0.06 | -0.11 | 0.00 | 0.03 | .071 |
| IV: Physical activity measured 120–150 mins before; DV: PA, NA and DS | | | | | | |
| PA | 1532 | 0.05 | 0.00 | 0.11 | 0.03 | .035 |
| NA | 1349 | -0.09 | -0.15 | -0.04 | 0.03 | .002 |
| DS | 1329 | -0.08 | -0.14 | -0.03 | 0.03 | .005 |
| IV: Physical activity measured 90–120 mins before; DV: PA, NA and DS | | | | | | |
| PA | 1581 | 0.03 | -0.02 | 0.08 | 0.03 | .213 |
| NA | 1399 | -0.06 | -0.11 | -0.01 | 0.03 | .031 |
| DS | 1377 | -0.06 | -0.12 | -0.01 | 0.03 | .031 |
| IV: Physical activity measured 60–90 mins before; DV: PA, NA and DS | | | | | | |
| PA | 1634 | 0.03 | -0.02 | 0.07 | 0.02 | .733 |
| NA | 1455 | -.01 | -0.06 | 0.04 | 0.03 | .767 |
| DS | 1429 | -0.02 | -0.07 | 0.03 | 0.03 | .733 |
| IV: Physical activity measured 30–60 mins before; DV: PA, NA and DS | | | | | | |
| PA | 1685 | 0.02 | -0.03 | 0.06 | 0.02 | .492 |
| NA | 1500 | -0.03 | -0.08 | 0.02 | 0.03 | .334 |
| DS | 1471 | -0.05 | -0.10 | 0.00 | 0.03 | .182 |
| IV: Physical activity measured 0–30 mins before; DV: PA, NA and DS | | | | | | |
| PA | 1744 | 0.03 | -0.01 | 0.08 | 0.02 | .167 |
| NA | 1557 | -0.05 | -0.10 | 0.00 | 0.03 | .099 |
| DS | 1524 | -0.07 | -0.12 | -0.02 | 0.03 | .021 |
| IV: PA, NA and DS; DV: Physical activity measured 0–30 mins after | | | | | | |
| PA | 1709 | 0.02 | -0.03 | 0.06 | 0.02 | .471 |
| NA | 1521 | -0.02 | -0.07 | 0.03 | 0.03 | .471 |
| DS | 1486 | -0.03 | -0.08 | 0.02 | 0.03 | .471 |
| IV: PA, NA and DS; DV: Physical activity measured 30–60 mins after | | | | | | |
| PA | 1623 | 0.03 | -0.02 | 0.07 | 0.02 | .522 |
| NA | 1436 | 0.00 | -0.05 | 0.05 | 0.03 | .885 |
| DS | 1401 | -0.02 | -0.07 | 0.03 | 0.03 | .522 |
| IV: PA, NA and DS; DV: Physical activity measured 60–90 mins after | | | | | | |
| PA | 1585 | 0.02 | -0.03 | 0.07 | 0.03 | .708 |
| NA | 1400 | -0.01 | -0.06 | 0.04 | 0.03 | .708 |
| DS | 1364 | -0.02 | -0.07 | 0.04 | 0.03 | .708 |
| IV: PA, NA and DS; DV: Physical activity measured 90–120 mins after | | | | | | |
| PA | 1546 | 0.02 | -0.02 | 0.07 | 0.03 | .946 |
| NA | 1361 | 0.00 | -0.05 | 0.05 | 0.03 | .946 |
| DS | 1327 | 0.00 | -0.05 | 0.05 | 0.03 | .946 |
| IV: PA, NA and DS; DV: Physical activity measured 120–150 mins after | | | | | | |
| PA | 1514 | 0.00 | -0.06 | 0.05 | 0.03 | .865 |
| NA | 1330 | -0.03 | -0.08 | 0.02 | 0.03 | .618 |
| DS | 1297 | -0.02 | -0.08 | 0.03 | 0.03 | .618 |
| IV: PA, NA and DS; DV: Physical activity measured 150–180 mins after | | | | | | |
| PA | 1459 | -0.01 | -0.07 | 0.04 | 0.03 | .594 |
| NA | 1282 | 0.02 | -0.03 | 0.08 | 0.03 | .594 |
| DS | 1249 | 0.01 | -0.04 | 0.07 | 0.03 | .594 |

*Note. n* = number of observations; *β* = standardized beta of the fixed effect; *LL* = lower limit of the confidence interval; *UL* = upper limit of the confidence interval; *SE* = standard error of the fixed effect; *p* = adjusted *p*-value.

**Supplementary Table 4**

*Results of multilevel models in which physical activity and the interactions between physical activity and low vs. high depressive symptom groups predicted the subsequent PA, NA, and DS, and models in which PA, NA, and DS and the interactions between PA, NA, or DS and low vs. high depressive symptom groups predicted the subsequent physical activity in all participants. Only the results of the interaction were presented.*

|  | *n* | β | *LL* | *UL* | *SE* | *p* |
| --- | --- | --- | --- | --- | --- | --- |
| IV: Physical activity measured 150–180 mins before; DV: PA, NA and DS | | | | | | |
| PA | 2565 | -0.02 | -0.10 | 0.06 | 0.04 | .959 |
| NA | 2248 | 0.00 | -0.09 | 0.08 | 0.04 | .959 |
| DS | 2217 | -0.02 | -0.10 | 0.07 | 0.04 | .959 |
| IV: Physical activity measured 120–150 mins before; DV: PA, NA and DS | | | | | | |
| PA | 2651 | 0.02 | -0.05 | 0.10 | 0.04 | .829 |
| NA | 2329 | 0.04 | -0.04 | 0.12 | 0.04 | .829 |
| DS | 2296 | 0.01 | -0.08 | 0.09 | 0.04 | .880 |
| IV: Physical activity measured 90–120 mins before; DV: PA, NA and DS | | | | | | |
| PA | 2737 | 0.08 | 0.00 | 0.16 | 0.04 | .112 |
| NA | 2413 | -0.05 | -0.13 | 0.03 | 0.04 | .260 |
| DS | 2376 | -0.07 | -0.15 | 0.01 | 0.04 | .144 |
| IV: Physical activity measured 60–90 mins before; DV: PA, NA and DS | | | | | | |
| PA | 2807 | 0.06 | -0.02 | 0.13 | 0.04 | .134 |
| NA | 2487 | -0.10 | -0.17 | -0.02 | 0.04 | .024 |
| DS | 2446 | -0.11 | -0.19 | -0.03 | 0.04 | .020 |
| IV: Physical activity measured 30–60 mins before; DV: PA, NA and DS | | | | | | |
| PA | 2895 | 0.04 | -0.03 | 0.12 | 0.04 | .279 |
| NA | 2570 | -0.05 | -0.13 | 0.03 | 0.04 | .279 |
| DS | 2524 | -0.06 | -0.14 | 0.02 | 0.04 | .279 |
| IV: Physical activity measured 0–30 mins before; DV: PA, NA and DS | | | | | | |
| PA | 2987 | 0.12 | 0.05 | 0.19 | 0.04 | .003 |
| NA | 2660 | -0.08 | -0.16 | -0.01 | 0.04 | .036 |
| DS | 2609 | -0.09 | -0.17 | -0.01 | 0.04 | .036 |
| IV: PA, NA and DS; DV: Physical activity measured 0–30 mins after | | | | | | |
| PA | 2912 | 0.10 | 0.03 | 0.18 | 0.04 | .018 |
| NA | 2584 | -0.07 | -0.15 | 0.01 | 0.04 | .074 |
| DS | 2536 | -0.09 | -0.17 | -0.01 | 0.04 | .037 |
| IV: PA, NA and DS; DV: Physical activity measured 30–60 mins after | | | | | | |
| PA | 2782 | 0.06 | -0.02 | 0.13 | 0.04 | .436 |
| NA | 2456 | 0.00 | -0.08 | 0.08 | 0.04 | .993 |
| DS | 2407 | -0.03 | -0.11 | 0.05 | 0.04 | .680 |
| IV: PA, NA and DS; DV: Physical activity measured 60–90 mins after | | | | | | |
| PA | 2720 | -0.01 | -0.09 | 0.06 | 0.04 | .940 |
| NA | 2397 | 0.02 | -0.07 | 0.10 | 0.04 | .940 |
| DS | 2347 | 0.00 | -0.09 | 0.08 | 0.04 | .940 |
| IV: PA, NA and DS; DV: Physical activity measured 90–120 mins after | | | | | | |
| PA | 2654 | 0.03 | -0.05 | 0.11 | 0.04 | .703 |
| NA | 2331 | -0.01 | -0.09 | 0.07 | 0.04 | .827 |
| DS | 2283 | -0.03 | -0.11 | 0.05 | 0.04 | .703 |
| IV: PA, NA and DS; DV: Physical activity measured 120–150 mins after | | | | | | |
| PA | 2602 | 0.05 | -0.03 | 0.13 | 0.04 | .429 |
| NA | 2282 | -0.03 | -0.11 | 0.06 | 0.04 | .518 |
| DS | 2235 | -0.05 | -0.13 | 0.04 | 0.04 | .429 |
| IV: PA, NA and DS; DV: Physical activity measured 150–180 mins after | | | | | | |
| PA | 2499 | 0.07 | -0.01 | 0.15 | 0.04 | .148 |
| NA | 2190 | -0.09 | -0.17 | -0.01 | 0.04 | .103 |
| DS | 2143 | -0.06 | -0.15 | 0.02 | 0.04 | .148 |

*Note. n* = number of observations; *β* = standardized beta of the fixed effect; *LL* = lower limit of the confidence interval; *UL* = upper limit of the confidence interval; *SE* = standard error of the fixed effect; *p* = adjusted *p*-value.

**Supplementary Table 5**

*Results of multilevel models in which light physical activity (LPA), moderate-to-vigorous physical activity (MVPA), and total physical activity (TPA) predicted the subsequent PA, NA, and DS in all participants (N = 78). Only the results of LPA were presented.*

|  | *n* | β | *LL* | *UL* | *SE* | *p* |
| --- | --- | --- | --- | --- | --- | --- |
| IV: Physical activity measured 150–180 mins before; DV: PA, NA and DS | | | | | | |
| PA | 4079 | 0.01 | 0.00 | 0.02 | 0.00 | .390 |
| NA | 3690 | 0.00 | -0.02 | 0.01 | 0.01 | .390 |
| DS | 3616 | 0.00 | -0.02 | 0.01 | 0.01 | .390 |
| IV: Physical activity measured 120–150 mins before; DV: PA, NA and DS | | | | | | |
| PA | 4078 | 0.01 | 0.00 | 0.02 | 0.00 | .018 |
| NA | 3689 | -0.01 | -0.02 | 0.00 | 0.01 | .031 |
| DS | 3615 | -0.01 | -0.02 | 0.00 | 0.01 | .018 |
| IV: Physical activity measured 90–120 mins before; DV: PA, NA and DS | | | | | | |
| PA | 4077 | 0.00 | -0.01 | 0.01 | 0.00 | .938 |
| NA | 3688 | 0.00 | -0.01 | 0.01 | 0.01 | .759 |
| DS | 3614 | 0.00 | -0.01 | 0.01 | 0.01 | .759 |
| IV: Physical activity measured 60–90 mins before; DV: PA, NA and DS | | | | | | |
| PA | 4074 | 0.01 | 0.00 | 0.02 | 0.00 | .015 |
| NA | 3686 | -0.02 | -0.03 | -0.01 | 0.01 | .003 |
| DS | 3612 | -0.02 | -0.03 | -0.01 | 0.01 | .001 |
| IV: Physical activity measured 30–60 mins before; DV: PA, NA and DS | | | | | | |
| PA | 4074 | 0.01 | 0.00 | 0.02 | 0.00 | .325 |
| NA | 3685 | 0.00 | -0.01 | 0.01 | 0.01 | .349 |
| DS | 3611 | -0.01 | -0.02 | 0.00 | 0.01 | .321 |
| IV: Physical activity measured 0–30 mins before; DV: PA, NA and DS | | | | | | |
| PA | 4071 | 0.01 | 0.00 | 0.02 | 0.00 | .220 |
| NA | 3682 | 0.00 | -0.01 | 0.01 | 0.00 | .698 |
| DS | 3608 | -0.01 | -0.02 | 0.00 | 0.01 | .220 |

*Note. n* = number of observations; *β* = standardized beta of the fixed effect; *LL* = lower limit of the confidence interval; *UL* = upper limit of the confidence interval; *SE* = standard error of the fixed effect; *p* = adjusted *p*-value.

**Supplementary Table 6**

*Results of multilevel models in which light physical activity (LPA), moderate-to-vigorous physical activity (MVPA), and total physical activity (TPA) predicted the subsequent PA, NA, and DS in all participants (N = 78). Only the results of MVPA were presented.*

|  | *n* | β | *LL* | *UL* | *SE* | *p* |
| --- | --- | --- | --- | --- | --- | --- |
| IV: Physical activity measured 150–180 mins before; DV: PA, NA and DS | | | | | | |
| PA | 4079 | 0.00 | 0.00 | 0.01 | 0.00 | .308 |
| NA | 3690 | 0.00 | -0.01 | 0.00 | 0.00 | .318 |
| DS | 3616 | -0.01 | -0.01 | 0.00 | 0.00 | .308 |
| IV: Physical activity measured 120–150 mins before; DV: PA, NA and DS | | | | | | |
| PA | 4078 | 0.00 | 0.00 | 0.01 | 0.00 | .415 |
| NA | 3689 | -0.01 | -0.01 | 0.00 | 0.00 | .415 |
| DS | 3615 | 0.00 | -0.01 | 0.00 | 0.00 | .415 |
| IV: Physical activity measured 90–120 mins before; DV: PA, NA and DS | | | | | | |
| PA | 4077 | 0.01 | 0.00 | 0.02 | 0.00 | .022 |
| NA | 3688 | -0.01 | -0.02 | 0.00 | 0.00 | .010 |
| DS | 3614 | -0.01 | -0.02 | 0.00 | 0.00 | .010 |
| IV: Physical activity measured 60–90 mins before; DV: PA, NA and DS | | | | | | |
| PA | 4074 | 0.00 | 0.00 | 0.01 | 0.00 | .330 |
| NA | 3686 | 0.00 | -0.01 | 0.00 | 0.00 | .433 |
| DS | 3612 | 0.00 | -0.01 | 0.00 | 0.00 | .330 |
| IV: Physical activity measured 30–60 mins before; DV: PA, NA and DS | | | | | | |
| PA | 4074 | 0.00 | 0.00 | 0.01 | 0.00 | .169 |
| NA | 3685 | -0.01 | -0.02 | 0.00 | 0.00 | .046 |
| DS | 3611 | -0.01 | -0.02 | 0.00 | 0.00 | .015 |
| IV: Physical activity measured 0–30 mins before; DV: PA, NA and DS | | | | | | |
| PA | 4071 | 0.01 | 0.00 | 0.02 | 0.00 | .013 |
| NA | 3682 | -0.01 | -0.02 | 0.00 | 0.00 | .013 |
| DS | 3608 | -0.01 | -0.02 | 0.00 | 0.00 | .011 |

*Note. n* = number of observations; *β* = standardized beta of the fixed effect; *LL* = lower limit of the confidence interval; *UL* = upper limit of the confidence interval; *SE* = standard error of the fixed effect; *p* = adjusted *p*-value.

**Figure S1.**

*The standardized betas and confidence intervals of multilevel models including an interaction between the main predictor and a dummy-coded group variable*


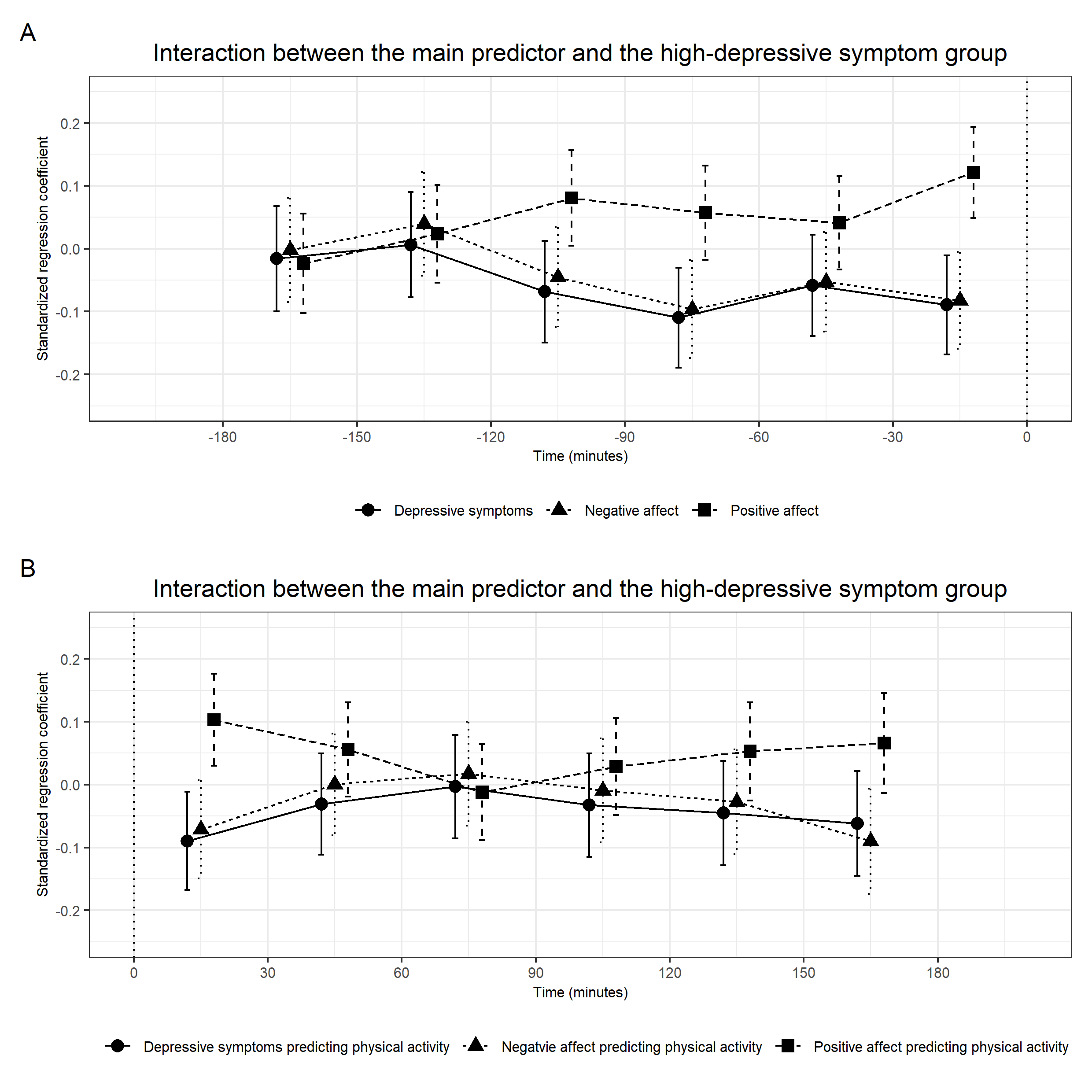


*Note.* Models where physical activity, a dummy-coded group variable, and the interaction between physical activity and the group variable predicted subsequent positive affect, negative affect, and depressive symptoms (A) and models where positive affect, negative affect, and depressive symptoms and dummy-coded group variable, and the interaction between mood and the group variable predicted subsequent physical activity (B).
